# Investigating the enhanced Best Performance Algorithm for Annual Crop Planning problem based on economic factors

**DOI:** 10.1371/journal.pone.0180813

**Published:** 2017-08-08

**Authors:** Aderemi Oluyinka Adewumi, Sivashan Chetty

**Affiliations:** School of Mathematics, Statistics and Computer Science, University of Kwa-Zulu Natal, Durban, South Africa; Lanzhou University of Technology, CHINA

## Abstract

The Annual Crop Planning (ACP) problem was a recently introduced problem in the literature. This study further expounds on this problem by presenting a new mathematical formulation, which is based on market economic factors. To determine solutions, a new local search metaheuristic algorithm is investigated which is called the enhanced Best Performance Algorithm (eBPA). eBPA’s results are compared against two well-known local search metaheuristic algorithms; these include Tabu Search and Simulated Annealing. The results show the potential of the eBPA for continuous optimization problems.

## Introduction

At present, the world is facing great challenges of both water scarcity and food supply shortages. Water scarcity is the occurrence when demands on fresh water availability exceed its supply [1]. The ever increasing world population growth contributes to this problem. As a result, greater demands on fresh water supply from all sectors of the industry is experienced. Major industry consumers of fresh water supply include the agricultural, domestic and industrial sectors. The more fresh water supply consumed by other sectors of the industry, the less will be made available for agricultural consumption. In spite of this present challenge, the agricultural sector—being the most important sector in that it is the primary producer of food globally—is now placed under pressure to use fresh water supplies more conservatively [1].

Currently, the estimation is that around 70% of fresh water supply globally is used by the agricultural sector. Of this, around 90% is estimated to be of consumptive use [1]. Therefore, if there is a reduction in the volume of fresh water that is supplied to the agricultural sector, the sustainability of food production will be threatened.

Specifically concerning crop production, fresh water supply is essential in order to achieve optimal crop growth, which is necessary in order to achieve maximum yield. Thus, water shortages in crop production will negatively affect crop growth, which in turn will affect harvests, which in turn will affect food supplies. Food supply shortages would result in increased food prices making it more costly to afford food. This will add to further socio-economic problems.

Therefore, to try and alleviate these challenges, it is imperative that the agricultural sector determine scalable solutions to the problem of resource allocations in crop production. In spite of the shortages of resources available, more returns are expected due to the increases in population growth.

As part of the attempt to contribute to the solution, this research concentrates on a crop planning problem known as the Annual Crop Planning (ACP) problem. The ACP problem was previously introduced in the literature by the same author of this paper [2, 3, 4,5]. Being focused at irrigation scheme level, the scope of the ACP problem is resource allocation solutions in annual crop planning.

Notably, no optimal solutions are guaranteed in crop planning. This is due to the uncertainties of several factors that are associated with crop production. Uncertain factors include, amongst others, those of climatic conditions, soil characteristics, cultivation practices, and the market demand and supply conditions determined within deregulated marketing environments. The aim of ACP solutions is to advise crop planners concerning resource allocations for the forthcoming crop production year.

Interesting studies on crop and irrigation planning include those by Mohamad and Said [6], Sunantara and Rimirez [7], Wardlaw and Bhaktikul [8], Georgiou and Papamichail [9], Sarker and Ray [10], Adeyemo and Otieno [11], Adeyemo *et al* [12], Pant *et al* [13], Pant *et al* [14], Raju and Kumar [15] and Reddy and Kumar [16]. Descriptions of these articles are also given in the studies by Chetty and Adewumi [2, 3, 4, 5].

This paper further expounds on the ACP problem. The ACP problem is reformulated in considering two fundamental market economic factors, namely economy of scale and the demand and supply relations. Furthermore, this study seizes the opportunity to investigate the newly introduced enhanced Best Performance Algorithm (eBPA) [17]. The solutions determined by the eBPA will be compared against the well-known Tabu Search (TS) and Simulated Annealing (SA) algorithms. This follows similar pattern as for other related space allocation problems (see [18–22]).

The rest of this paper is structured as follows. Section 2 describes the economy of scale and the demand and supply relational factors. These will be implemented as part of the new ACP mathematical model which is presented in section 3. Section 4 describes the metaheuristic algorithms. Section 5 presents the case study. Section 6 describes the experimental results. Finally, section 7 draws conclusions and outlines possible future work.

## Economy of scale and demand and supply relations

The economy of scale and the demand and supply relations have always had a notable presence in crop production. With the economy of scale influence, crop production on a larger scale has always been more profitable as unit costs are lower [23]. Especially with the advent of farming technologies such as machinery, fertilizers, irrigation practices, etc., the economy of scale influence in crop production has been considerable. Almost every aspect of modern crop production favours production on a larger scale. Concerning the market demand and supply factors in crop production, the sale of the harvests are done within deregulated marketing environments. Therefore, in an environment where there are no governmental control over the market prices, the market prices are determined by demand and supply relations.

Economy of scale is described as the reduction in the unit cost, per item being produced, as the volume of output increases [24]. This is well researched in market economics and could occur for several reasons: the fixed costs per unit decrease as the volume of the items produced increase (for example, the fixed cost of South Africa Rand (ZAR) 100 is calculated to be cheaper per unit in producing 100 units as compared to 10. The resultant effect of this is increased profit earned per unit of the item produced); fixed costs per unit are calculated to be cheaper in purchasing materials in larger volumes at discounted prices; the utilization of specialized machinery in manufacture result in more efficiency per unit of production thus reducing costs; etc.

**Fig 1:**
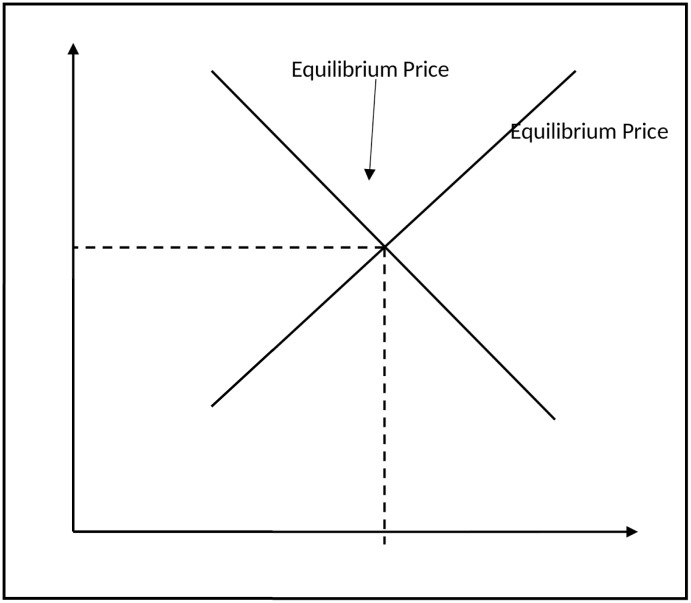
Equilibrium market price as determined by the demand and supply relations.

Demand and supply relations quantify the mathematical relations between the quantity of goods demanded by the buyers, and that supplied by the producers, at specific market prices (market price is also referred to as the “equilibrium price”) [25]. Hence, the demand relation refers to the quantity of goods demanded by buyers at the equilibrium price that they are willing to pay. Similarly, the supply relation refers to the supply of goods by producers at the equilibrium price that they are willing to supply at. The demand and supply relations therefore determine the equilibrium price as agreed upon by the buyer and the seller. In exercising the trade, producers will want to maximize their profit earned, while buyers will want to acquire the goods at the lowest possible price. An illustration of the demand and supply relations is given in Fig 1.

In Fig 1, *P* represents price and *Q* quantity. The equilibrium price is where *P* and *Q* intersect. This means that quantity *Q* will be traded at price *P*. At any price below *P*, the quantity of produce demanded will increase (due to a lower cost factor). On the other hand, at any price above *P*, the demand will decrease due to the reluctance to purchase at higher prices.

The ACP mathematical model presented in section 3 incorporates these important economic factors; it is necessary in order to determine realistic solutions.

## ACP mathematical model with economic factors for an existing irrigation scheme

This section presents the new ACP mathematical model which includes the market economic factors of economy of scale and the demand and supply relations. Explanations on the foundational ACP mathematical models are found in Chetty and Adewumi [2, 3, 4, 5]. The mathematical model in this study relate to that of an existing irrigation scheme.

To implement the economy of scale influence, a “fixed cost” variable is introduced. Hence, production costs are now explicitly differentiated as being fixed and variable. A fixed cost factor associated with the production of each crop will encourage a higher quantity of produce, as the unit cost will decrease and will result in higher profit earned per crop. However, this influence is challenged by the demand relational factor, in that higher yields beyond equilibrium price will result in lower producer prices which equates to less profit earned per unit (and vice versa).

In this model, equilibrium price is represented in terms of hectare allocations in using either of the demand or supply relational equations. Hence, with gross profits earned being dependant on hectare allocations, it is now interesting that hectare allocations and gross profits are influenced by the economy of scale and the demand and supply relational factors. This introduces added complexity, yet allows for more realistic solutions.

The ACP mathematical model, with market economic factors, is as follows:

### Indices

- *k*—Plot types. (1 = single-crop plots; 2 = double-crop plots; 3 = triple-crop plots; etc.).
- *i*—Indicative of the crop groups that are grown in sequence of each other on the same farming plot of land within the year, on plot type *k* (*i* = 1 indicates the 1^st^ crop group; *i* = 2 indicates the 2^nd^ crop group; *i* = 3 indicates the 3^rd^ crop group; etc.).
- *j*—Indicative of the individual crops belonging to crop group *i*, on plot *k*.

### Input parameters

- *l*—Number of different farming plot types.
- *N*_*k*_—Number of sequential crop groups cultivated on plot *k*.
- *M*_*ki*_—Number of individual crops cultivated at stage *i*, on plot *k*.
- *H*_*kij*_—Hectare allocation (ha) of crop *j*, at stage *i*, on plot *k* as determined from the previous year.
- *L*_*ki*_—Total area of land, in hectares (ha), allocated for crop production at stage *i*.
- *FR*_*kij*_—Average fraction per hectare of crop *j*, at stage *i*, on plot *k*, which needs to be irrigated (1 = 100% coverage, 0 = 0% coverage).
- *R*_*kij*_—Averaged rainfall estimates, in meters (m), that fall during the growing months for crop *j*, at stage *i*, on plot *k*.
- *CWR*_*kij*_—Crop water requirements, in meters (m), of crop *j*, at stage *i*, on plot *k*.
- *A*—Volume, in m^3^, of irrigated water that can be supplied per hectare (ha^-1^).
- *P*—Price of irrigated water m^-3^.
- *O*_*kij*_—Operational cost ha^-1^ of crop *j*, at stage *i*, on plot *k*. This cost excludes the cost of irrigated water per crop.
- *F*_*kij*_—Fixed cost of production for crop *j*, at stage *i*, on plot *k*.
- *YD*_*kij*_—The expected yield in tons per hectare (t ha^-1^) of crop *j*, at stage *i*, on plot *k*.
- *MP*_*kij*_—Producer price per ton of crop produced for crop *j*, at stage *i*, on plot *k*. This is the equilibrium price from the previous year of trading, at the hectares allocated. It is determined by the demand/supply relation.
- *Lb*_*kij*_—Lower bound of crop *j*, at stage *i*, on plot *k*. This reflects the minimum expected market demand, in hectares (ha).
- *Ub*_*kij*_—Upper bound of crop *j*, at stage *i*, on plot *k*. This reflects the maximum expected market demand, in hectares (ha).

### Calculated parameters

The calculation of the following parameters are required in advance before determining solutions. *TA* is required for Eq 3.4 below. *IR*_*kij*_ is required in order to calculate *C_IR*_*kij*_. *C_IR*_*kij*_ is required in order to calculate *C*_*kij*_. Finally, *C*_*kij*_ is required for the *AV*_*kij*_ calculation found under the “variables” section.

- *TA*—Total volume of irrigated water that can be supplied to the total area of farming land within the year (*TA* = *T* * *A*).
- *IR*_*kij*_—Volume of irrigated water that should be supplied to crop *j*, at stage *i*, on plot *k*. (*IR*_*kij*_*m*^3^ = (*CWR*_*kij*_*m* − *R*_*kij*_*m*) * 10000*m*^2^ * *FR*_*kij*_).
- *C_IR*_*kij*_—The cost of irrigated water ha^-1^ of crop *j*, at stage *i*, on plot *k*. (*C_IR*_*kij*_ = *IR*_*kij*_ * *P*).
- *C*_*kij*_—Variable cost ha^-1^ of crop *j*, at stage *i*, on plot *k*. (*C*_*kij*_ = *O*_*kij*_ + *C_IR*_*kij*_).

### Variables

- *X*_*kij*_—Area of land, in hectares, that can be feasibly allocated for the production of crop *j*, at stage *i*, on plot *k*.
- *AV*_*kij*_—Average cost ha^-1^ in considering the fixed and variable costs of production for crop *j*, at stage *i*, on plot *k*. (*AV*_*kij*_ = (*X*_*kij*_*C*_*kij*_ + *F*_*kij*_)/*X*_*kij*_).
- *EP*_*kij*_—Equilibrium price that is substituted by using either the demand or supply relations, which has dependency on *X*_*kij*_ (e.g. Demand relation: *X*_*kij*_(D) = a + b*EP*_*kij*_; Supply relation: *X*_*kij*_(S) = c + d*EP*_*kij*_ where a, b, c and d are constants).

### Objective function

$$\begin{array}{ll} \text{Maximize }f & =\sum_{k=1}^{l}\sum_{i=1}^{N_{k}}\sum_{j=1}^{M_{ki}}X_{kij}(EP_{kij}*YD-AV_{kij})\\ & =\sum_{k=1}^{l}\sum_{i=1}^{N_{k}}\sum_{j=1}^{M_{ki}}X_{kij}(EP_{kij}*YD-C_{kij})-F_{kij} \end{array}$$ (3.1)

Eq 3.1 gives the objective function. The fixed cost variable *F*_*kij*_ implements the economy of scale influence. The equilibrium price variable *EP*_*kij*_ is used to implement the market demand/supply influence; *EP*_*kij*_ is substituted in terms of hectare allocations by using either of the demand or supply equations. The constraints to the problem remain the same, yet for convenience is given below.

### Land allocation constraints

Feasible solutions must satisfy the lower and upper bound constraints of each crop.

$$Lb_{kij}\leq X_{kij}\leq Ub_{kij}\;\forall k,i,j$$ (3.2)

The summation of the area of land allocated for the production of each crop *j*, at stage *i*, on plot *k*, must not exceed the total area of land available for crop production at stage *i*, on plot *k*.

$$\sum_{j}^{M_{ki}}X_{kij}\leq L_{ki}\;\forall k,i$$ (3.3)

### Irrigated water constraints

The summation of the volume of irrigated water allocated for the production of each crop must be less than the total volume that can be supplied to the irrigation scheme within the year.

$$\sum_{k}^{}\sum_{i}^{}\sum_{j}^{}IR_{kij}\leq TA$$ (3.4)

### Non-negative constraints

Arbitrarily, the lower and upper bound settings, as well as the gross profits earned per crop must be non-negative.

$$Lb_{kij},\;Ub_{kij},\;(EP_{kij}*YD-AV_{kij})>0\;\forall k,i,j$$ (3.5)

## Local search algorithms

The first ACP mathematical model was introduced in Chetty and Adewumi [2, 3]. This research constituted determining solutions for existing irrigation schemes. The second ACP mathematical model was introduced in Chetty and Adewumi [3, 4]. This research constituted determining solutions at a new irrigation scheme. For both problem instances, population-based and local search metaheuristic algorithms were investigated. These included the Cukoo Search, the Firefly Algorithm, Glow-worm Swarm Optimization, the Genetic Algorithm, SA and TS.

For the ACP mathematical model presented in this paper, only SA, TS and the eBPA will be investigated. Reason being, this study constitutes yet initial research into the potential of the eBPA. Furthermore, eBPA is designed based on similar underlying principles implemented by both SA and TS.

The motivation for the development of the eBPA was in realizing that there are apparent weaknesses in the strategic designs of SA and TS. With TS, it was realized that although it employs the benefit of memory strategies, it lacks in its stochastic ability. On the other hand, although SA is pure stochastic, the disadvantage is that it does not employ memory strategies and hence loses valuable solutions found during its search trajectory. The development of the eBPA was thus an attempt to bridge the strength of the memory ability of TS and the stochastic ability of SA. Below are given brief descriptions on each algorithm.

### Simulated annealing

Briefly, SA [26, 27] is modeled on the analogy of the atomic composition of metal. At higher temperatures, the atomic composition of metal is more volatile. Yet, it will stabilize as the metallic structure begins to cool. Stability (or equilibrium) is reached at a temperature close to zero. For the annealing process to be successful, the decrease in the rate of temperature must be slow. Volatility represents SA’s ability to accept worst solutions. It is represented with probability *P* = *exp*[(*C*–C*)/*T*], where *C* is the cost of the current solution, C* is the cost of the candidate solution, and *T* is the temperature. At higher temperatures, the probability of accepting worst solutions is higher. This allows SA to explore different neighborhood regions of the solution space with more ease. Using this strategy, more promising neighborhood regions can be located. However, as the temperature decreases, this probability also decreases and there is a transition from exploration to exploitation. Greater levels of exploitation presents SA the opportunity to concentrate on those promising neighborhood regions found in trying to identify high quality solutions. The greatest levels of exploitation are achieved at very low temperatures where the probability of accepting worst solutions are at its lowest. The strategy of accepting worst solutions is two-fold: new regions are explored, and a doorway is presented to escape local entrapment. With SA, significant research has been done around the setting of its parameter values, which significantly influences the performance of the algorithm. The initial temperature (*T*) importantly controls the transition from exploration to exploitation, and the cooling factor (*a*) importantly controls the rate at which the algorithm converges to its final solution.

### Tabu search

TS is based on the analogy of something that should not be touched or interfered with [28, 29]. This is achieved by maintaining a limited number of recently found best candidate solutions in a list called the Tabu List (*TL*). The *TL* is commonly implemented in a first-in-first-out (FIFO) way. Candidate solutions are determined in searching the neighborhood region of current solution *x*, i.e. *N*(*x*). Therefore, the maximum number of candidate solutions considered will be *N*(*x*) − |*TL*|, as any solution recorded in the *TL* has a tabu status and will not be interfered with. The decision to reject the *TL* solutions minimizes the risk of cycling. Thus, TS makes use of memory in intelligently directing the search.

### The enhanced Best Performance Algorithm

The eBPA is modeled on the analogy of professional athletes desiring to improve upon their best registered performances within competitive environments. The strategy employed by the eBPA is to maintain a limited number of the best performances delivered by an athlete in a list called the Performance List (*PL*). The athlete then tries to improve upon these performances by learning from the strengths and weakness of the performances registered in the *PL*. The smaller the size of the *PL* the more difficult it will be to register further improved performances.

There are six foundational rules governing the design of the eBPA:

1. An athlete maintains an archive of a collection of a limited number of best performances.
2. From this collection, the record of the worst performance is identified. This becomes the minimum benchmark standard for the athlete to try and improve upon.
3. If a new performance is delivered which improves upon (or is at least equivalent to) that of the worst performance, then the archive is updated by replacing the performance of the worst with that of the new. However, upon performing the update, if it is realized that the result of the new performance is identical to that of any other performance in the archive, but different in terms of the technique that had been employed, then the new performance will replace the one with the identical result.
4. The athlete will continue to try and improve upon the performance that caused the most recent update of the archive.
5. All performances registered in the archive must be unique in terms of result and technique.
6. The archive size is strategically reduced until only one performance remains.

To artificially simulate this analogy, eBPA distinguishes the *best* and *worst* solutions in the *PL*. The *working* solution is the one indexed as the next solution to be worked with.

To try and improve upon the *worst* solution registered in the *PL*, local search moves will be applied to a copy of the *working* solution; hence, a new solution *working*′ will be realized. *Working*′ is chosen from the candidate list of neighboring solutions related to *working*. If *working*′ at least improves upon *worst* (or is at least equivalent in solution quality, yet unique in terms of its design variables compared to that of *worst*) then the *PL* will be updated by replacing *worst* with *working*′. Being newly inserted into the *PL*, *working*′ will then become the next *working* solution. Also, if *working*′ has improved upon best, then it will be indexed as the new *best* solution. The *worst* solution thereafter would need to be re-determined (i.e. re-indexed). The new *worst* solution will now be of an improved benchmark standard if the solution qualities of the *worst* and *working*′ solutions were not identical. If an update of the *PL* has not been made, then local search moves will continue to be applied to a copy of *working*. However, given a certain probabilistic factor, the next *working* solution could be that of *working*′. The probabilistic factor represents the desire of the athlete to try out a new technique. However, this will continue indefinitely as determined by the probabilistic factor.

**Fig 2:**
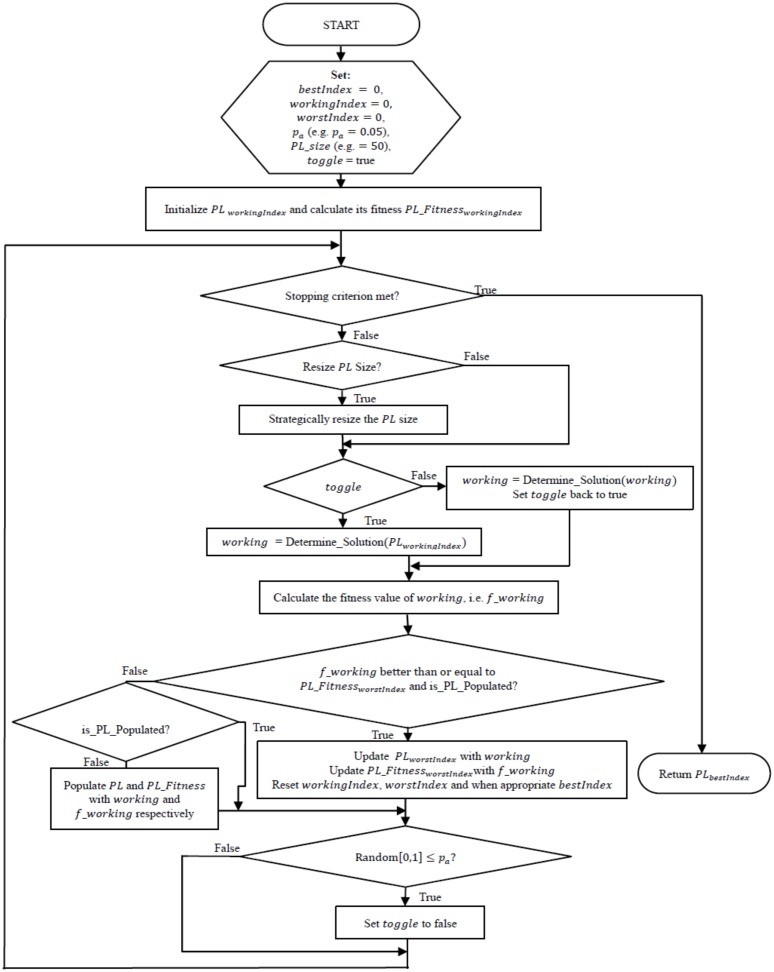
Flowchart diagram of the eBPA.

After the termination criterion is satisfied, the *best* solution will be returned. This solution is representative of the best performance given off by the athlete. eBPA is presented in Fig 2 below. In Fig 2, “is_PL_populated” checks to see if the memory structure has been fully populated, and if not then populate it with *working*′.

## The vaalharts irrigation scheme case study

The dataset studied are those of the nine crops studied in Chetty and Adewumi [2, 4]. It relates to the Vaalharts Irrigation Scheme (VIS) located in South Africa. Comprising of approximately 36,950 hectares of prime agricultural land, the VIS is one of the largest irrigation schemes in the world [30,31,32].

The geographical region of the VIS is known for cold and frosty winters, warm summers and irregular rainfall patterns. With irregular rainfall patterns, and having a low rainfall average of 440 millimeters (mm) annum^-1^, irrigated water is necessary for optimized crop production at the VIS. Table 1 below shows the average rainfall patterns as determined over a period of 36 years.

**Table 1:** Mean rainfall statistics, in millimeters (mm), as determined over a 36 year period [31,32].

	Jan	Feb	Mar	Apr	May	Jun	Jul	Aug	Sep	Oct	Nov	Dec
Mean Rainfall	75.9	63.5	71.8	51.6	19.9	9.5	4.3	8.6	11.3	24.6	45.7	58.0

The irrigated water supplied to the farm plots get extracted from the nearby Vaal River. It is supplied at a maximum rate of 9,140 m^3^ ha^-1^ annum^-1^. A water charge of 8.77 cents m^-3^ needs to be paid to the Vaalharts Water User Association (WUA) [31,32].

Table 2 shows the statistics of the primary crops grown at the VIS. The table lists the crop names, together with their types given in brackets; these crop types are either perennial (p), summer (s) or winter (w) crops. The table also gives the hectare allocations per crop (ha’s crop^-1^), the tons of yield per hectare (t ha^-1^), the Crop Water Requirements (CWR’s) in millimeters (mm), the average rainfall statistics (AR) in millimeters (mm), the producer prices per ton of yield (ZAR t^-1^), the average fraction of irrigated water applied per hectare per crop, with *FR*_*kij*_ ∈ [0, 1], the cost of the irrigated water per hectare (*C_IR*_*kij*_), and the operational costs of production per crop (*O*_*kij*_). From Table 2 it is calculated that the total area of land for the perennial, summer and winter crops are 8,300 ha’s, 15,500 ha’s, and 12,200 ha’s respectively.

**Table 2:** Dataset for the vaalharts irrigation scheme case study [31,32].

Crops	ha’s crop^-1^	t ha^-1^	CWR	AR	ZAR t^-1^	^*FR*^_*kij*_	*C_IR*_*kij*_	*O*_*kij*_
Pecan Nuts (p)	100	5.0	1,600	444.7	3,500.00	1	1,013.20	5,833.35
Wine Grapes (p)	300	9.5	850	350.8	2,010.00	1	437.80	6,365.00
Olives (p)	400	6.0	1,200	444.7	2,500.00	1	662.40	4,999.98
Lucerne (p)	7,500	16.0	1,445	444.7	1,185.52	1	877.26	6,322.72
Cotton (s)	2,000	3.5	700	386.4	4,500.00	1	275.03	5,250.00
Maize (s)	6,500	9.0	979	279.0	1,321.25	1	613.90	3,963.78
Ground Nuts(s)	7,000	3.0	912	339.5	5,076.00	1	502.08	5,076.00
Barley (w)	200	6.0	530	58.3	2,083.27	1	413.68	4,166.52
Wheat (w)	12,000	6.0	650	58.3	2,174.64	1	518.92	4,349.28

## Experimental results

### Experimental data

Table 3 gives the lower and upper bound settings, the fixed costs of production (*F*_*kij*_), as well as the demand equations used for the experiment. For the purpose of simulation, demand equations were formulated for each crop using the statistics of the equilibrium price ton^-1^ of yield (i.e. the *MP*_*kij*_), and the hectares allocated (i.e. the *H*_*kij*_).

**Table 3:** Parameter settings per crop.

Crops	*Lb*_*kij*_	*Ub*_*kij*_	*F*_*kij*_ (ZAR)	*EP*_*kij*_ (Demand Eq.)
Pecan Nuts (p)	50	300	875,000	30*X + 500
Wine Grapes (p)	100	500	2,864,250	5*X + 510
Olives (p)	100	800	2,700,000	7*X– 300
Lucerne (p)	7,000	8,000	948,416	(2/5)*X + 1814.48
Cotton (s)	1,000	3,000	393,750	2*X + 500
Maize (s)	5,000	8,000	8,323,875	X/4–303.75
Groundnuts (s)	4,500	9,500	1,522,800	X/2 + 1576
Barley (w)	100	300	7,249,779.6	10*X + 83.27
Wheat (w)	10,000	15,000	1,565,740.8	X/6 + 174.64

### Simulation strategy

The parameter settings of metaheuristic algorithms influence their performance per problem instance. Therefore, for fair algorithmic comparisons for this problem instance, experiments will be performed to determine the appropriate parameter settings for each metaheuristic algorithm. Determining the parameter settings will be the first set of experiments. Once the parameter setting for the algorithms have been determined, the second set of experiments will be performed for the algorithmic comparisons.

For problem instances where the optimal solution is known, the objective in comparing algorithmic performances is to monitor which algorithm will determine the optimal solution in the shortest computational time. Therefore, with this being the intent, the parameter settings would need to be adjusted accordingly. Another alternative in comparing algorithmic performances is to run simulations for a fixed number of iterations. With this approach, the parameter settings would need to be adjusted to make the most effective use of the limited computational time available. One possible problem with this approach is that if the metaheuristic algorithm shows a clear convergence, in leading towards its best solution, this strategy would be ineffective if the termination were to be done before this point of convergence. Therefore, for these reasons, the stopping criterion adopted in this study is to execute termination of the algorithms at their points of convergence.

Convergence is the point where further improvements in the solution quality would yield minimal benefits, compared to the relatively large number of iterations required to yield those minimal benefits. Therefore, in this study, convergence will be detected when no further improved best solution is found for a large number of iterations. For the experiments to determine the parameter settings, a total of 30,000 idle iterations will be used to detect convergence. Thereafter, in comparing algorithmic performances, a total of 50,000 idle iterations will be used to detect convergence.

### Experiment 1: Determination of parameter settings

#### eBPA parameter settings

**Fig 3:**
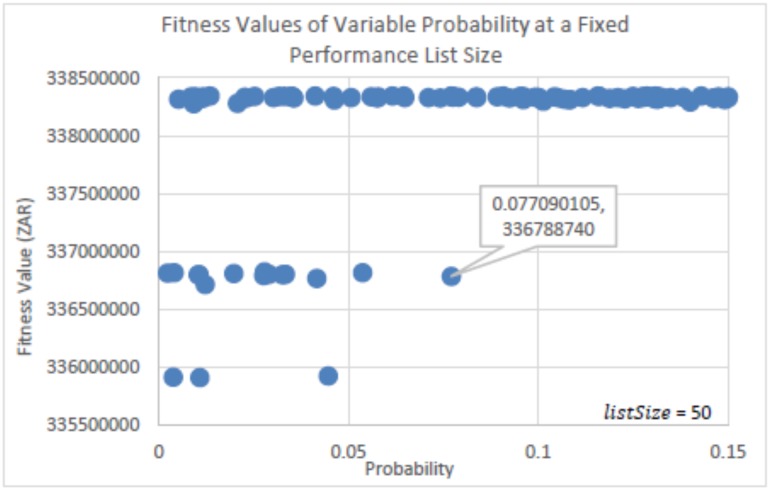
Fitness values determined using randomly selected probability factors, at a fixed performance list size of 50.

**Fig 4:**
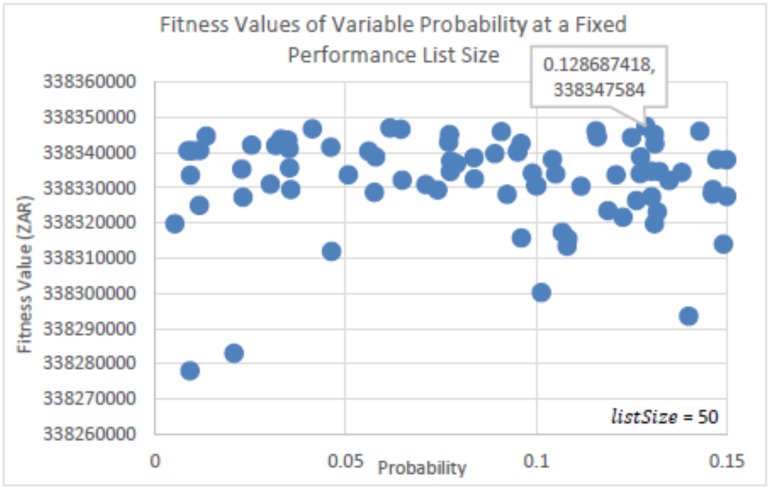
Zoomed in image of Fig 3.

**Fig 5:**
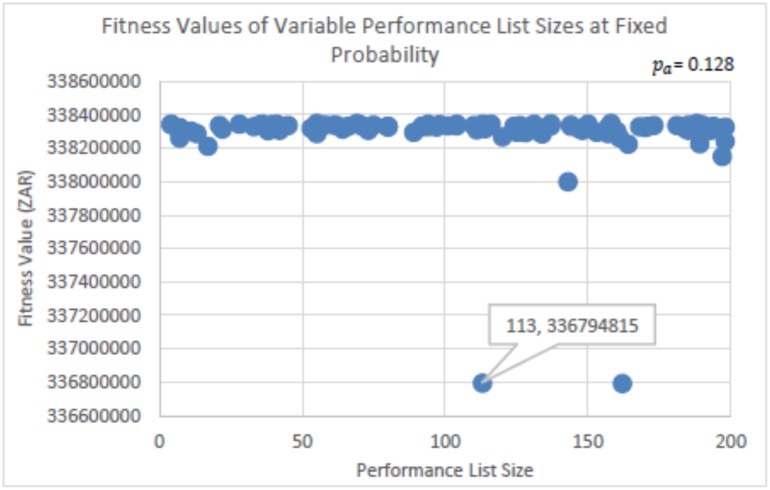
Fitness values determined using randomly selected Performance List sizes at a fixed probability factor of 0.128.

**Fig 6:**
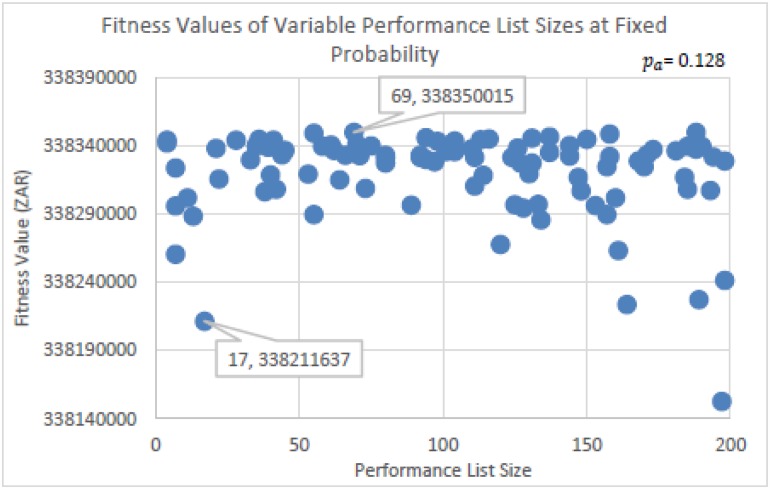
Zoomed in image of Fig 5.

The experiments run to determine the parameter settings for the probability factor (*p*_*a*_) and the Performance List size (*listSize*) of eBPA is seen in Figs 3, 4, 5 and 6. In Fig 3, *listSize* remained fixed at 50, while *p*_*a*_ was randomly selected from within the range of 0 < *p*_*a*_ ≤ 0.15. This was per run for a total of 100 runs, using the same initial solution. Fig 4 is a zoomed in image of Fig 3, and shows more clearly the best solutions determined. Fig 3 shows that with probability factors below 0.078, many solutions were determined that were found in regions that were far away from those of the best solutions found. However, it is seen that there is no distinguished best value for *p*_*a*_, as competitive solutions can be seen scattered throughout the probability range. This shows that irrespective of the value of *p*_*a*_, eBPA would find good neighborhood regions, yet with more consistency if the probability factor were greater than 0.077. The best solution determined, as seen in Fig 4, had a probability factor of 0.128 (truncated to three decimal places). Therefore, for the rest of the experiments, the value of *p*_*a*_ = 0.128 will be used.

For the experiments run to determine the Performance List size, the value of *p*_*a*_ = 0.128 remained fixed, while the value of *listSize* was randomly selected from within the range of 1 ≤ *listSize* ≤ 200. Again, this was per run for a total of 100 runs in using the same initial solution. The results are seen in Figs 5 and 6. Fig 6 is a zoomed in image of Fig 5. From Figs 5 and 6, it is seen that the most consistent performances were determined using *listSize*’s within the range of 18 and 112. However, it is again observed that eBPA determined competitive solutions throughout the Performance List size range. The best solution had a *listSize* of 69, which will be used for the algorithmic performance comparison tests.

With the termination criterion to be set at 50,000 idle iterations, the strategy to be used to reduce of the Performance List size, until a size of 1 is reached, will be as follows: If half of the termination number of idle iterations has been reached (i.e. 25,000 = *minimum_condition* = *termination_criterion*/2), divide the remaining number of iterations by the current Performance List size (i.e. *reduction_Criterion* = (*termination_criterion*–*minimum_condition*)/*listSize*). If the lower bound plus the reduction criterion (i.e. *minimum_condition* + *reduction_Criterion*) equates to the current number of idle iterations, then reduce the Performance List size by 1. The reduction of the Performance List size has the dual purpose of increasing the level of exploitation with matured search, as well as reducing the risk of cycling.

#### Simulated annealing parameter settings

**Fig 7:**
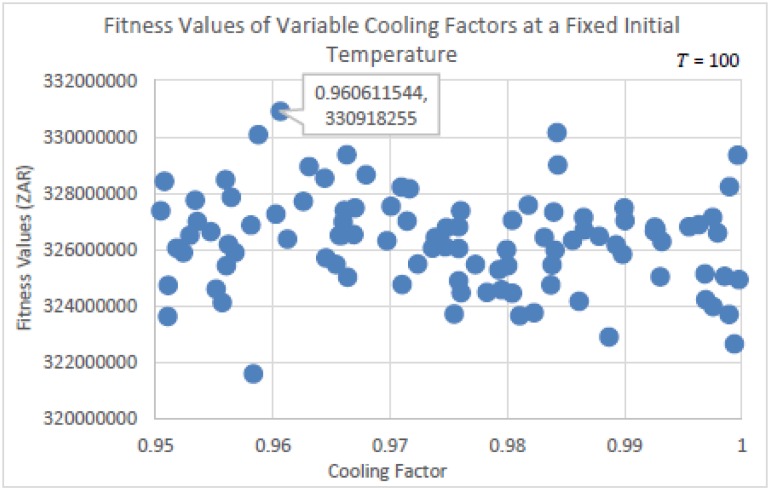
Fitness values determined using randomly selected cooling factors, at a fixed initial temperature of 50.

**Fig 8:**
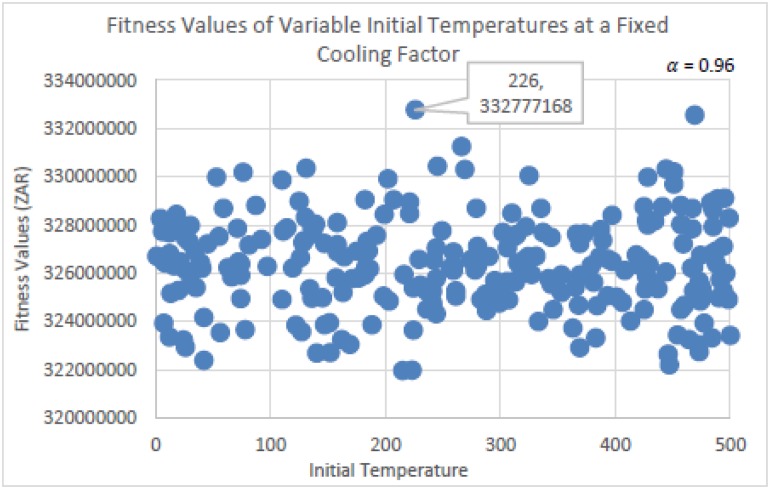
Fitness values determined using randomly selected initial temperature values, at a fixed cooling factor of 0.96.

The experiments run to determine the parameter settings for SA are seen in Figs 7 and 8 below. In Fig 7, the initial temperature *T* was fixed at 100, while the cooling factor *a* had been randomly selected from within the range of 0.95 ≤ *a* < 1. This was done per run for a total of 100 runs in using the same initial solution. The cooling factor *a* controls the rate of convergence, and decreases *T* using the equation *T* = *T* * *a*. Therefore, the higher the value of *a*, the slower will be the rate of convergence, and the more successful will be the annealing process. From Fig 7, it is observed that the fitness qualities of the solutions were similar in having found similar neighborhood regions. The best value of *a* seen is 0.96 (rounded off to two decimal places).

The value of *a* = 0.96 remained fixed for the experiment related to Fig 8, while the initial temperature *T* was randomly selected from within the range of 1 ≤ *T* ≤ 500. This was done per run for a total of 250 runs in using the same initial solution. More runs were needed to determine *T*, as *T* importantly controls the transition from exploration to exploitation. The parameter settings for SA are more difficult to determine, and would explain the volume of research done on SA. From Fig 8, it is seen that the best solution for *T* was 226. Together with *a* = 0.96, these are the parameter settings that will be used for SA in performing the algorithmic comparison tests.

#### Tabu search parameter settings

**Fig 9:**
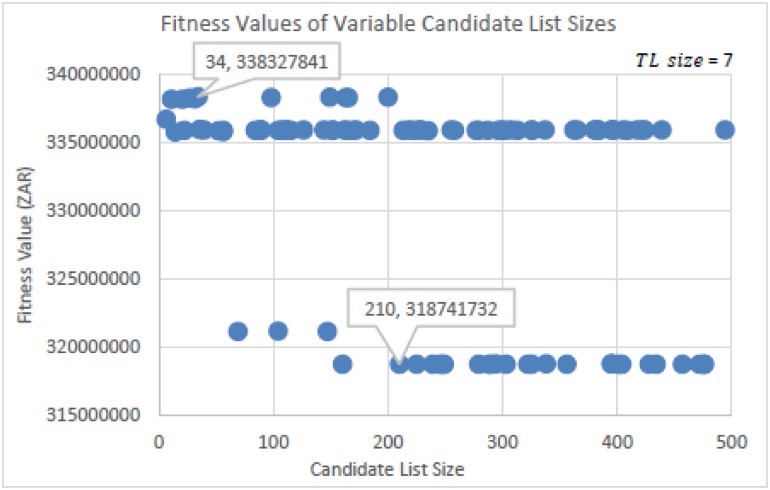
Fitness values determined by randomly selecting the Candidate List size values.

**Fig 10:**
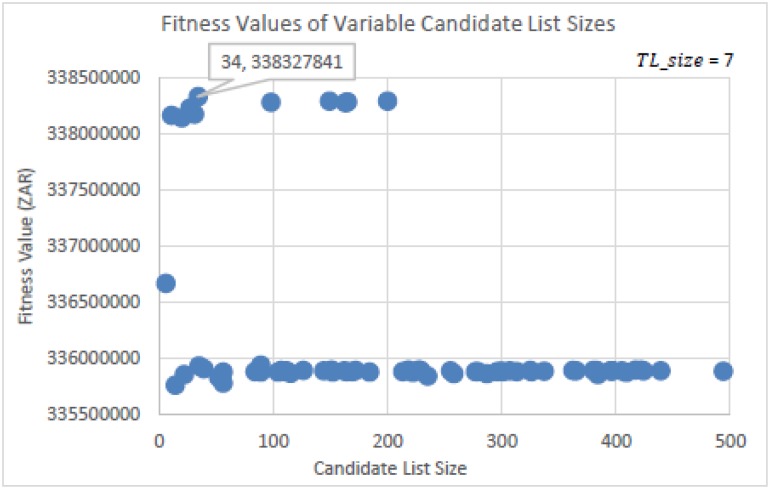
Zoomed in image of Fig 9.

The experiments run to determine the Candidate List size (*CL_size*) for TS is seen in Figs 9 and 10. Fig 10 is a zoomed in image of Fig 9. For this experiment, a recommended Tabu list size (*TL_size*) of 7 was used by Glover [28,29, 33]. *CL_size*’s were randomly selected from within the range of 1 ≤ *CL_size* ≤ 500. This was done per run for a total of 100 runs in using the same initial solution. Fig 9 shows that *CL_size*’s above 209 determined solutions that had fitness values which were far from the best solution found. The best solution found, as seen more closely in Fig 10, had a *CL_size* of 34. Fig 10 also shows a cluster of competitive solutions found around the *CL_size* of 34. This indicates that a size of 34 is a good value to choose. These values are the parameter settings that will be used for TS in performing the algorithmic comparison tests.

#### Summary of experiment 1

As can be seen from Figs 3 and 5, the parameter settings for eBPA did not significantly hinder is performances. This is an interesting observation in being compared to an algorithm such as SA which requires more effort to set its parameter values. The benefit would be seen if the parameter values of both algorithms remained the same in running different problem instances. For example, running different instances of the Travelling Salesman Problem.

The summary of the parameter settings is seen in Table 4 below.

**Table 4:** Summary of the parameters settings used for experiment 2.

Method	Parameters	
eBPA	Probability (*p*_*a*_)	0.128
	Performance List Size (*listSize*)	96
SA	Initial temperature (*T*)	226
	Alpha (*a*)	0.96
TS	Tabu List Size (*TL_size*)	7
	Candidate List Size (*CL_size*)	34

### Experiment 2: Algorithmic performance comparisons

For the second experiment, in comparing the algorithmic performances, the parameter settings determined from the first set of experiments were used. For this experiment, a total of 50 runs per metaheuristic algorithm were executed. The termination criterion was 50,000 idle iterations. For each of the 50 runs, per algorithm, the same initial randomly generated solution was passed in as an input parameter to each algorithm. The experiments performed, together with these test criterion, were sufficient to ensure fair algorithmic comparisons. From the 50 solutions determined by each algorithm, their overall best and average solutions are documented. Their 95% Confidence Interval (CI) values are also documented for their fitness values.

In Table 5, the average execution times give an indication of the number of best solutions found by each metaheuristic algorithm. Reason being, each time the best solution had been improved upon, the counter for the idle number of iterations had been reset and consequently resulted in an increase in the execution time. As can be observed, eBPA’s spent more time on average searching for solutions. This means that the eBPA intelligently found more promising neighborhood regions within the solution space compared to TS and SA.

**Table 5:** Average execution time performances (AVG) in milliseconds (ms).

Methods	AVG (ms)
eBPA	148,178
TS	52,367
SA	33,029

Table 6 gives the statistical values of the overall best (BFV) and average (AFV) fitness value solutions. The 95% CI values are also given, along with the initial solution. The fitness value refers to the total gross profit earned.

**Table 6:** Statistics of the best and average fitness values solutions, along with the 95% CI values.

Methods	BFV (ZAR)	AFV (ZAR)	95% CI
Initial Solution	290,775,157	N/A	N/A
eBPA	338,351,684	338,345,193	AFV ± 1,203
TS	338,340,881	337,493,100	AFV ± 261,742
SA	330,721,884	327,791,514	AFV ± 425,002

It is observed that each algorithm determined best solutions that improved upon the initial solution. eBPA determined the best BFV and AFV solutions, and had the lowest 95% CI value. This was followed by TS and then SA. eBPA’s best solution determined a gross profit of ZAR 10,803, ZAR 7,629,800 and ZAR 47,576,527 more than that of TS, SA and the initial solution respectively. On average, eBPA performed significantly better than TS. eBPA also showed more consistency in having a smaller 95% CI estimate. Having determined the best BFV and AFV solutions, along with the lowest 95% CI value concludes that eBPA was the strongest and most consistent algorithm for this problem instance.

The strength of eBPA is attributed to the techniques employed in maintaining the improved solutions registered in its memory structure called the Performance List (*PL*). The *PL* maintains a limited number of the best solutions found (at any given time) while traversing through the solution space. This maintenance is based on the idea of allowing solutions that meet the minimum criterion to be allowed acceptance into the *PL*. The minimum criterion is that the fitness value of the worst solution must at least be met or improved upon. If the fitness value of the worst solution has been met, then the design variables of the new solution must be unique to be allowed acceptance. Upon performing the update, the indices referencing the *best*, *working* and *worst* solutions need to be re-determined. These techniques, along with the probability factor used to try and escape local entrapment, and the reduction of the *PL* size, show to be an effective blend in traversing the solution space quickly yet determining high quality solutions. This observation is made in comparing eBPA’s solutions with that of TS and SA for this difficult optimization problem. The techniques employed by the eBPA finds an intermediatory point between the memory search technique employed by TS, and the single-point stochastic search technique employed by SA.

Table 7 gives the statistical values of the irrigated water requirements (IWR), and that of the costs of production (CP). As can be observed, each algorithm determined improved irrigated water allocation solutions over that of the initial solution. Interestingly, the costs of production values were also lower though the gross profit margins were higher. From all algorithms, eBPA determined a solution that required the least volume of irrigated water. eBPA determined a solution that required a volume of 2,493,689 m^3^ less than that of the initial solution. This was followed by TS, which required a volume of 2,492,815 m^3^ less. SA required a volume of 1,730,665 m^3^ less. These solutions conform to the objective of yielding higher returns per unit of irrigated water consumed. At the quota of 9,140 m^3^ha^-1^annum^-1^, these savings would be able to supply irrigated water to an additional 272.8, 272.7 and 189.3 hectares of agricultural land by eBPA, TS and SA respectively.

**Table 7:** Statistical values of the irrigated water requirements (IWR) and the costs of production (CP).

Methods	IWR (m^3^)	CP (ZAR)
Initial Solution	244,491,000	156,924,202
eBPA	241,997,311	154,799,423
TS	241,998,185	154,799,348
SA	242,760,335	154,985,403

**Fig 11:**
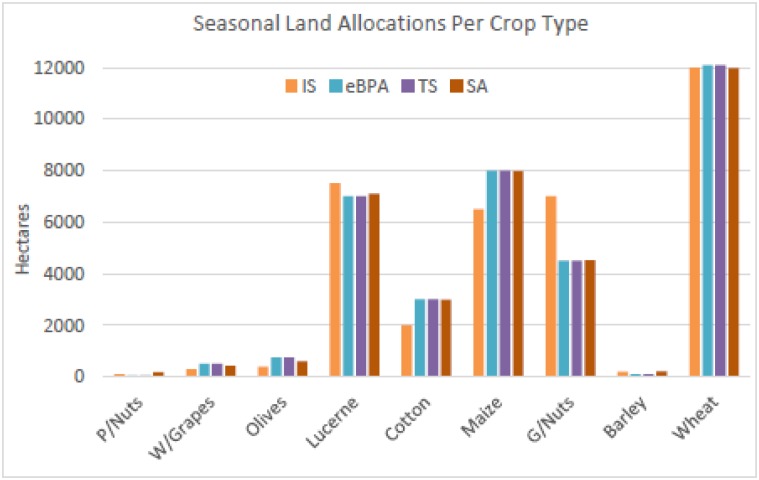
Comparison of the hectare allocation solutions per crop.

Fig 11 shows graphical comparisons of the hectare allocation solutions. eBPA and TS show to have determined similar solutions. The metaheuristic solutions are also seen to be comparable to that of the initial solution due to the constraints of the lower and upper bound settings.

The statistics of the hectare allocations (ha’s crop^-1^), irrigated water requirements (IWR), and the costs of production (CP) of the initial and that of the best metaheuristic solutions are seen in Table 8 below.

**Table 8:** Statistics of the initial (IS) and metaheuristic solutions per crop.

Crops	Methods	ha’s crop^-1^	IWR (m^3^)	CP (ZAR)
Pecan Nuts	IS	100	1,155,300	597,153.143
	eBPA	50.003	577,685.304	254,847.493
	TS	50.001	577,662.84	254,834.181
	SA	174.722	2,018,562.036	1,108,738.936
Wine Grapes	IS	300	1,497,600	1,849,889.52
	eBPA	499.995	2,495,977.51	3,210,418.552
	TS	499.751	2,494,757.158	3,208,755.529
	SA	430.796	2,150,534.609	2,739,669.671
Olives	IS	400	3,021,200	2,114,959.24
	eBPA	749.99	5,664,672.134	4,096,740.2
	TS	750.215	5,666,375.011	4,098,016.827
	SA	604.264	4,564,003.826	3,271,581.702
Lucerne	IS	7,500	75,022,500	40,722,449.25
	eBPA	7,000.012	70,021,117.62	37,122,515.7
	TS	7,000.033	70,021,327.18	37,122,666.53
	SA	7,090.218	70,923,452.63	37,772,005.34
Cotton	IS	2,000	6,272,000	9,475,054.4
	eBPA	2,999.988	9,407,960.899	15,000,012.71
	TS	2,999.828	9,407,459.508	14,999,129.36
	SA	2,987.453	9,368,653.092	14,930,759.94
Maize	IS	6,500	45,500,000	23,809,100
	eBPA	7,999.944	55,999,604.87	30,675,316.6
	TS	7,999.779	55,998,450.03	30,674,561.4
	SA	7,986.315	55,904,203.44	30,612,928.84
Ground Nuts	CP	7,000	40,075,000	32,193,977.5
	eBPA	4,500.069	25,762,894.54	18,249,155.67
	TS	4,500.394	25,764,754.36	18,250,967.76
	SA	4,526.232	25,912,678.59	18,395,095.89
Barley	IS	200	943,400	791,047.98
	eBPA	100.001	471,707.002	333,032.689
	TS	100.001	471,703.748	333,029.529
	SA	224.294	1,057,994.541	902,319.617
Wheat	IS	12,000	71,004,000	45,370,570.8
	eBPA	12,099.999	71,595,691.22	45,857,383.66
	TS	12,099.999	71,595,695.3	45,857,387.02
	SA	11,975.706	70,860,252.72	45,252,302.99

The program was written in the Java programming language. It was programmed using the Netbeans^®^ 7.0 Integrated Development Environment. All simulations where run on the same platform. The computer used had a Windows^®^ 7 Professional operating system, an Intel^®^ Core™ i5 Processor, 8 GB of RAM and a 500GB hard-drive.

## Conclusion

This study further expounds on the recently introduced Annual Crop Planning (ACP) problem in the literature. In this study, a new mathematical formulation for the ACP problem is introduced. It is based on the market economic factors of economy of scale and the demand and supply relations. The objective of the ACP problem is optimized resource allocation solutions in crop production. This study is motivated by increased concerns of water scarcity, and other limited resources available for crop production. In spite of the limited resources available for crop production, more output is required per unit due to increases in food demand. The ACP problem is a relevant problem within the agricultural sector.

In determining solutions, a new local search metaheuristic algorithm in the literature is investigated. It is called the enhanced Best Performance Algorithm (eBPA). eBPA’s solutions were compared against those of Tabu Search (TS) and Simulated Annealing (SA). To ensure fairness in performing algorithmic comparisons, experiments were run to determine the appropriate parameter settings for each metaheuristic algorithm. The termination criterion for the algorithms was a fixed number of idle iterations. This represented the point of convergence for each algorithm. The results show that the techniques employed by the eBPA are effective in having determined the overall best solutions.

eBPA shows good potential as an alternative algorithm for difficult optimization problems. An added benefit of the eBPA is the simplicity in setting its parameter values. This paper constitutes yet initial study into the potentials of the eBPA. Further study is required to test the potentials of the eBPA to other types of optimization problems.
